# Research on total factor energy efficiency in western China based on the three-stage DEA-Tobit model

**DOI:** 10.1371/journal.pone.0294329

**Published:** 2024-04-16

**Authors:** Lingshu Zhang, Jina Cui

**Affiliations:** School of Finance and Economics, Qinghai University, Xining City, Qinghai Province, China; Sichuan University, CHINA

## Abstract

As an essential material basis and power source for economic and social development, Western China’s low energy use efficiency has hindered its sustainable economic development. This study aims to evaluate the total factor energy efficiency of the region and identify its influencing factors. A three-stage DEA model was used to measure the efficiency of 11 provinces from 2006 to 2021, and the Tobit model was employed to investigate internal factors. The findings show that (i) external environmental factors and stochastic perturbations have a significant impact on TFEE in the western region, overestimating integrated efficiency and scale efficiency and underestimating pure technical efficiency. (ii) the study of external influencing factors finds that the level of economic development increases input redundancy; the industrial structure increases capital input and labor input redundancy while decreasing energy input redundancy; and the energy consumption structure increases capital input and energy input redundancy while decreasing labor input redundancy. (iii) the study of internal influencing factors finds that the level of scientific and technological innovation, the level of openness to the outside world, and the TFEE have a positive correlation. In contrast, the intensity of environmental regulation has a negative correlation.

## Introduction

China has recognized the urgent need to address the environmental crisis and reduce the consumption of resources and energy. In 2020, the country pledged to achieve a carbon peak by 2030 and carbon neutrality by 2060. Energy is crucial in driving economic and social development, which is vital to achieving these goals. The Chinese Government has prioritized the issue of energy. It has emphasized the need for significant improvements in energy resource utilization and the promotion of green and low-carbon industrial production methods in its 14th Five-Year Plan. Promoting energy efficiency is essential for achieving sustainable economic growth and realizing China’s "double carbon" goal.

The western region of China is a vast area that makes up 71% of the country’s total area. It is rich in energy resources. However, due to technological and managerial limitations, the energy utilization efficiency in the region could be higher. This hampers sustainable economic development and industrial transformation. The Blue Book of the West: Report on the Development of Western China (2021) highlights that low productivity, low energy input-output ratios, and a low level of regional industrial structure are the main characteristics of economic development in the Western region. What factors impact this region’s total energy efficiency (TFEE), and how can policies be formulated to enhance it, facilitate sustainable economic development, and promote industrial upgrading? It is necessary to scientifically assess the TFEE and the factors that influence it in the Western region. The assessment findings can provide a scientific basis for local governments to draft relevant policies.

Currently, the methods for measuring TFEE are divided into parametric and non-parametric methods. Parametric methods are represented by stochastic frontier analysis. Still, due to its need to consider the specific form of the production function, the basic assumptions are more complex and prone to bias problems. In contrast, non-parametric methods represented by data envelopment analysis can avoid the problem. However, most existing studies use the traditional DEA model, ignoring the influence of external environmental factors and random interference on the efficiency assessment. Fried proposed a three-stage DEA model to eliminate external environmental factors and random interference to make the measurement results more accurate [1]. In addition, the current measure of TFEE excludes too many environmental factors. It ignores exploring its internal influencing factors, which cannot provide a more reliable basis for the subsequent government policy design. Based on this, this paper firstly adopts a three-stage DEA model to measure TFEE in the western region to solve the inaccuracy of energy efficiency measurement caused by external environmental factors and stochastic disturbances; and secondly, uses the Tobit model to explore further the internal factors affecting TFEE in the western region, to provide references for the design of government policies.

The marginal contributions of this paper are as follows: (1) Adopting the three-stage DEA model to overcome the influence of external environmental factors and random interference on energy efficiency measurement in the traditional DEA model and to measure the TFEE in the western region more scientifically and accurately. (2) Considering the separability of the influencing factors, the Tobit model is used to explore further the influence of the level of scientific and technological innovation, environmental regulation, and the level of opening up to the outside world on the TFEE to avoid neglecting the exploration of the internal influencing factors due to the exclusion of too many external environmental factors.

The study is divided into several parts. The second part provides a literature review, while the third part discusses the research and data methodology used in this paper. The fourth part presents the measured results of TFEE, and the fifth part explores its internal influences. Finally, the sixth part offers conclusions, discussions of this research, and recommendations.

## Literature review

Energy efficiency refers to the degree to which the actual energy input is optimal for a given output or the degree to which the actual maximum production is achieved for a given energy input. Hu and Wang proposed the concept of total factor energy efficiency to calculate energy efficiency in a total factor framework and explore the relationship between multifactor inputs and outputs [2]. The concept of TFEE has been widely used in research. Currently, the TFEE research mainly focuses on the decomposition of measurement and investigation of influencing factors.

The methods of measuring and decomposing TFEE are mainly divided into parametric and non-parametric methods, among which the non-parametric method is represented by the data envelopment analysis (DEA) method. The current research is primarily based on the DEA method and improved [3, 4].

Traditional DEA models ignore the effects of external environmental factors and random disturbances. Fried pointed out in his study that in the conventional DEA model, the performance of decision-making units has efficiency losses due to managerial inefficiency, environmental factors, and statistical noise, which should be eliminated [1]. In recent years, many studies have introduced three-stage DEA models to measure carbon emission efficiency, urbanization efficiency, technological innovation efficiency, and other issues [5–7]. However, there is still room for research on energy efficiency in the three-stage DEA model, so this paper chooses the three-stage DEA model to measure the TFEE of the 11 provinces in China’s western region from 2006 to 2021.

In the investigation of the influencing factors of TFEE, many papers have analyzed from different perspectives, such as the level of economic development, industrial structure, property rights structure, technology level, energy price, energy consumption structure, environmental regulation, and the level of openness [8–12]. At the same time, scholars have also analyzed the influencing factors from different perspectives. Yang et al. divide the factors influencing regional energy efficiency into endogenous economic and social factors and exogenous natural environmental factors [13]. Pan et al. believe that the influencing factors of energy efficiency can be divided into four categories: structural elements, technological factors, market factors, and human factors [14]. Gong divided the influencing factors into short-term and long-term factors from the time perspective [15]. Jin categorizes the influencing factors into innate and acquired factors, which consider innate factors as influencing factors beyond humans, while acquired factors have room for adjustment and improvement [16].

Total factor energy efficiency has been thoroughly studied and explored in the literature, but there is still room for further deepening. First, the current measurement of TFEE mostly uses the one-stage DEA model. Still, the use of this model ignores the interference of external factors and random interference, which makes the measurement results have errors. Secondly, the current studies do not categorize the influencing factors and exclude too many external factors without exploring the internal ones, which does not provide more references for subsequent policy design. Third, the current research on regional TFEE mainly focuses on the whole country, and some of the studies focusing on regions also concentrate on developed areas such as Eastern China. In contrast, the research in the West needs to be more extensive. This paper, therefore, endeavors to fill the above research gap.

## Research and data methodology

### Research methodology

**Fig 1:**
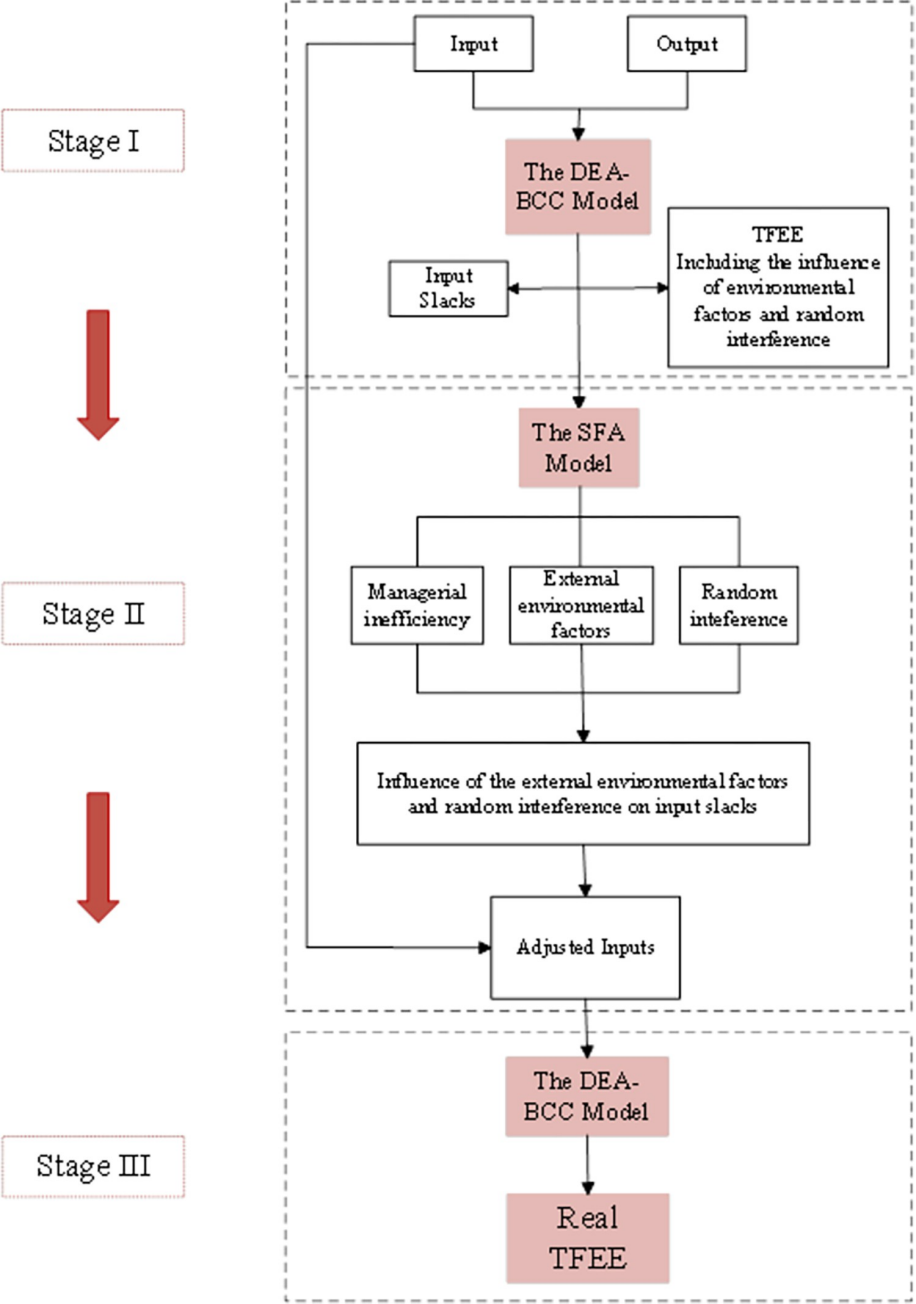
Three-stage DEA model.

The three-stage DEA model excludes the effects of managerial inefficiency, environmental factors, and statistical noise, making the measurements more accurate. In recent years, many studies have introduced three-stage DEA models to measure the efficiency of different objects. This paper draws on Wang and Luo, using the three-stage DEA model to measure the TFEE [17, 18]. The analysis framework is shown in Fig 1.

The first stage uses the original input and output factors for the initial efficiency measurement. Among them, the DEA model chooses the input-oriented variable payoff of scale (BCC) model. The BCC model in pairwise form under the input orientation of each decision unit is:

$$\min\;\theta -\varepsilon ({\hat{e}}^{T}S^{-}+e^{T}S^{+})$$

$$s.t.\left\{ \begin{array}{l} \sum_{j=1}^{n}X_{j}\lambda_{j}+S^{-}=\theta X_{0}\\ \sum_{j=1}^{n}Y_{j}\lambda_{j}-S^{+}=Y_{0}\\ \lambda_{j}\geq 0,S^{-},S^{+}\geq 0 \end{array}\right.$$ (1)

Where j = 1,2,⋯,*n* represent the decision unit. *N* represents the input factor, and *Y* represents the output factor.

The combined efficiency (TE) derived from the BCC model can be decomposed into pure technical efficiency (PTE) and scale efficiency (SE) with the following equations:

TE=PTE×SE (2)

The input factor slack variables capture the initial inefficiencies. The second stage decomposes the initial input factor slack variables into the three effects mentioned above. It regresses the environmental variables and the mixed error term using the slack variables from the first stage. The aim is to eliminate the effects of the environment and randomness in the system on efficiency so that the decision units are coordinated in the same environment.

Using each input slack variable as the explanatory variable and external environmental factors as the explanatory variables, regression equations were constructed for each input slack variable as follows:

$$S_{ni}=f(Z_{i};\beta_{n})+\nu_{ni}+\mu_{ni}$$ (3)

i=1,2,⋯,I;n=1,2,⋯,N

Where *S*_*ni*_ denotes the slack value of the input factor; *Z*_*i*_ denotes the external environmental variables, and *β*_*n*_ is the coefficient of the environmental variable; *ν*_*ni*_+*μ*_*ni*_ is the mixed error term, and *ν*_*ni*_~*N*(0,*σ*_*ν*_^2^) denotes the effect of random interference on the input slack variables; *μ*_*ni*_ denotes the impact of management factors on the input slack variables, which are assumed to obey a normal distribution truncated at zero, *μ*~*N*^+^(0,*σ*_*μ*_^2^). Adjusting for the original input variables, the formula is as follows:

XniA=Xni+[max(f(Zi;β^n))−f(Zi;β^n)]+[max(νni)−νni] (4)

i=1,2,⋯,I;n=1,2,⋯,N

Where $X_{ni}^{A}$ is the adjusted input factor; *X*_*ni*_ is the original input before adjustment; [max(f(Zi;β^n))−f(Zi;β^n)] is an adjustment for external environmental factors; [*max*(*ν*_*ni*_)−*ν*_*ni*_] is to bring all decision units to the same level of luck. Random interference need to be separated from management inefficiency when performing on the original inputs, and mthe following equation estimates management inefficiency

$$E(\mu |\varepsilon )=\sigma_{*}\left[\frac{\phi (\lambda \frac{\varepsilon }{\sigma })}{\Phi (\frac{\lambda \varepsilon }{\sigma })}+\frac{\lambda \varepsilon }{\sigma }\right]$$ (5)

Among them, $\sigma_{*}=\frac{\sigma_{\mu }\sigma_{\nu }}{\sigma },\sigma =\sqrt{{\sigma_{\mu }}^{2}+{\sigma_{\nu }}^{2}},\;\lambda =\sigma_{\mu }/\sigma_{\nu }$。

The random interference term is calculated as follows:

$$E[v_{\mathrm{ni}}|v_{\mathrm{ni}}+\mu_{\mathrm{ni}}]=s_{\mathrm{ni}}‐f(z_{i};\beta_{n})-E[u_{\mathrm{ni}}|v_{\mathrm{ni}}+\mu_{\mathrm{ni}}]$$ (6)

The third stage reruns the DEA using the adjusted input factors and the original output variables. Environmental factors and randomness are excluded in this case, and the measured efficiency is more accurate.

### Variable description

The variables selected for the three-stage DEA model in this paper include input and output factors and external environmental factors. The input factors are energy, labor, and capital as indicators, and the variables are described as follows:

1. Energy input: The energy consumption of coal, oil, natural gas, and hydroelectricity is converted and summed up according to the corresponding ratio to represent the annual comprehensive energy consumption of each province, in million tons of standard coal;
2. Labor input: In measuring the labor force, many studies have chosen labor time and educational attainment as indicators of labor input. However, there are no official statistics on labor time in China [19]. In this paper, the number of people employed at the end of the year in each province is chosen as the labor input, in 10,000;
3. Capital input: Based on Shan Haojie’s study, each province’s capital stock is calculated using the perpetual inventory method [20]. The calculation formula is: *Cit* = *Ci,t*–1 (1 – *θ*) + *Iit*, where *Cit* is the capital stock in the year i of region t, θ is the depreciation rate, and *Iit* is the new investment in constant prices in the year i in region t. (b) Referring to the previous literature, the depreciation rate is set at 10%, the new investment is measured by the current investment in social fixed assets, and the capital stock of each province in the calendar year is converted at comparable prices in 2006, in billions of yuan;
4. Undesired factor: Based on the principle that undesired outputs are the same as inputs, the less the better. Regarding the studies of Pittman and Reinhard, this paper considers undesired outputs as a kind of input to be brought into the DEA model for investigation [21, 22]. Sulfur dioxide in exhaust gases is the focus of environmental monitoring in China. In this paper, Sulphur dioxide emissions are selected as the undesired output.
5. Desired output: Energy is an indispensable material basis for economic development and a driving force for economic growth. It is the driving force of economic growth and lays the material foundation for economic development. The total output that reflects economic growth is usually measured by gross domestic product (GDP). This paper uses the GDP of each province for the current year as the desired output. The base period of 2006 is used to convert to real GDP to ensure data consistency.

Environmental factors mainly refer to factors that have an impact on total factor energy efficiency but are not subjectively controlled. This paper relates to the classification method of Yang and Jin on the influencing factors of energy efficiency and considers the divisibility of influencing factors. It is believed that the influencing factors beyond humans should be selected as external environmental factors when eliminating environmental factors while retaining internal influencing factors that can be improved in the future and further exploring the degree of influence of internal factors in the subsequent study, which can better provide a reference for following improvement policies. Therefore, the external environmental factors considered in the second stage of DEA in this paper include: the level of economic development, industrial structure, and energy consumption structure, and the specific variables are described as follows:

1. Level of economic development: the level of regional economic development will directly affect the behavior and decision-making of regional economic actors. Consistent with other similar studies, this paper uses the GDP per capita of each province as an indicator of the level of economic development;
2. Industrial structure: A meaningful way to improve TFEE is to promote industrial transformation and upgrading. As the primary energy consumption industry in China, the development of the secondary industry has an essential impact on TFEE. Based on Wang’s research, this paper adopts the proportion of the added value of the secondary industry in the GDP of each province as an indicator of industrial structure [23];
3. Energy consumption structure: Different types of energy contribute differently to economic development, and the energy structure is an essential factor affecting energy efficiency. In China’s energy structure, coal is dominant. Based on Peng’s research, this paper adopts the proportion of coal consumption to energy consumption as an indicator to measure the energy consumption structure [24].

### Data source

This paper selects data from 11 provinces in western China from 2006–2021 as the decision unit for energy efficiency evaluation. Tibet Autonomous Region was not included in the analysis due to data availability. All research data were obtained from the China Statistical Yearbook, China Energy Statistical Yearbook, and statistical yearbooks of relevant provinces and regions. The descriptive statistics of each variable are shown in Table 1:

**Table 1:** Descriptive statistics for variables.

Variable	Minimum	Maximum	Mean	Standard deviation
GDP (billion yuan)	585.20	227510.89	33810.87	38421.13
Sulfur dioxide emissions (tonnes)	4.01	155.70	54.37	38.59
Capital (billion yuan)	4196.20	298542.61	63903.61	55306.27
Energy (million tonnes of standard coal)	277.00	4745.00	1834.63	1182.02
Labor force (million people)	2085.00	28478.28	9579.06	5084.50
Level of economic development	8.81	0.61	3.48	1.78
Industrial structure	0.54	0.31	0.42	0.05
Energy consumption structure	0.89	0.24	0.59	0.16

## The western region TFEE measurement results and comparative analysis

### Stage I: Traditional DEA empirical results

In this paper, we use Deap2.1 software to measure the total factor energy efficiency of eleven provinces in the western region of China using the input and output factors selected above substituted into the BCC model, and the results are shown in Table 2:

**Table 2:** Measured TFEE of the first phase in the western region by province, 2006–2021.

Year	Inner Mongolia	Guangxi	Chongqing	Sichuan	Guizhou	Yunnan	Shaanxi	Gansu	Qinghai	Ningxia	Xinjiang
2006	1.000	1.000	1.000	1.000	0.948	1.000	0.978	1.000	0.762	0.694	1.000
2007	1.000	1.000	1.000	1.000	0.982	1.000	0.978	1.000	0.767	0.747	1.000
2008	1.000	0.984	1.000	1.000	1.000	1.000	1.000	1.000	0.803	0.815	1.000
2009	1.000	0.970	1.000	1.000	1.000	1.000	1.000	0.950	0.761	0.817	0.945
2010	1.000	0.907	1.000	1.000	1.000	0.946	0.991	0.909	0.763	0.803	0.916
2011	1.000	0.813	1.000	1.000	1.000	0.926	0.931	0.832	0.691	0.754	0.865
2012	1.000	0.750	1.000	1.000	1.000	0.937	0.907	0.747	0.633	0.700	0.822
2013	0.965	0.748	1.000	1.000	1.000	0.956	0.863	0.685	0.596	0.666	0.789
2014	0.906	0.726	1.000	1.000	0.975	0.908	0.810	0.612	0.570	0.621	0.746
2015	0.867	0.743	1.000	1.000	0.972	0.862	0.738	0.528	0.558	0.581	0.661
2016	0.815	0.926	1.000	0.894	0.918	0.813	0.787	0.470	0.552	0.545	0.606
2017	0.758	0.895	1.000	1.000	0.905	0.807	0.786	0.458	0.535	0.551	0.597
2018	0.767	0.722	1.000	0.923	0.920	0.848	0.722	0.506	0.565	0.566	0.657
2019	0.761	0.617	1.000	0.920	0.904	0.856	0.710	0.529	0.560	0.588	0.656
2020	0.714	0.571	1.000	0.896	0.889	0.834	0.670	0.537	0.536	0.616	0.629
2021	0.780	0.573	1.000	0.877	0.856	0.802	0.681	0.556	0.526	0.659	0.652
Average value	0.896	0.809	1.000	0.969	0.954	0.906	0.847	0.707	0.636	0.670	0.784
Sequence	5	7	1	2	3	4	6	9	11	10	8

As can be seen from the results, when external environmental factors and random interference are not excluded, the average value of TFEE in the western region of China from 2006 to 2021 is 0.822. Specifically, Chongqing has an overall efficiency of 1, located in the efficiency frontier surface, while all other regions have room for improvement. Among them, the energy efficiency of Guizhou, Sichuan, Inner Mongolia, Yunnan, and Shaanxi is greater than the average value of the Western region. At the same time, Xinjiang, Guangxi, Gansu, Ningxia, and Qinghai are lower than the regional average, with more room for improvement.

Table 3 shows the results of the decomposition of TFEE for 11 provinces in the western region from 2006 to 2021 in the first stage. From the decomposition results, it is concluded that Chongqing is on the efficiency frontier in both pure technical efficiency and scale efficiency. Sichuan and Qinghai are on the efficiency frontier in pure technical efficiency. Besides, there is room for improvement in pure technical efficiency and scale efficiency in the rest of the provinces. Among them, Guangxi, Yunnan, Shaanxi, Gansu, and Xinjiang are below the average of the Western region in pure technical efficiency and have more room for improvement. In contrast, Gansu, Qinghai, and Ningxia have more room for improvement in scale efficiency.

**Table 3:** Decomposition results of TFEE in the first phase for each province in the western region.

Region	Comprehensive Efficiency	Pure Technical Efficiency	Scale Efficiency	Compensation for Size
Inner Mongolia	0.896	0.918	0.974	irs
Guangxi	0.809	0.890	0.905	irs
Chongqing	1.000	1.000	1.000	-
Sichuan	0.969	1.000	0.969	drs
Guizhou	0.954	0.981	0.972	irs
Yunnan	0.906	0.922	0.982	irs
Shaanxi	0.847	0.860	0.984	irs
Gansu	0.707	0.804	0.863	irs
Qinghai	0.636	1.000	0.636	irs
Ningxia	0.670	0.992	0.675	irs
Xinjiang	0.784	0.839	0.928	irs
Average Value	0.834	0.928	0.899	

Since the results of the first stage include the influence of external environmental factors and random interference, they cannot reflect each region’s actual level of total factor energy efficiency, so further adjustment and measurement are needed.

## Stage Ⅱ: SFA regression results

The input slack variables in the decision cell derived from the conventional DEA in the first stage were used as explanatory variables, respectively, and the economic development level, industrial structure, and energy consumption structure described above were used as explanatory variables in the regression using the software Frontier 4.1. The regression results were obtained as shown in Table 4 below:

**Table 4:** SFA regression results.

Variables	Capital Input	Labor Input	Energy Input	Sulfur Dioxide Input
Constant Term	-2583.23**	449.76**	-7301.083**	-6.03
	(492.36)	(195.44)	(1127.29)	(9.53)
Economic Development Level	667.74**	19.74*	803.14***	1.29**
	(178.58)	(10.99)	(59.36)	(0.56)
Industry Structure	-11091.53***	-580.03*	126.58	-1.94
	(307.92)	(429.32)	(2434.80)	(22.01)
Energy Consumption Structure	6034.15***	-671.43**	5863.03***	1.98
	(390.62)	(177.35)	(316.13)	(7.61)
σ^2^	34982384.00***	117353.35***	12118717.00***	195.17**
	(1.000)	(1.54)	(1.00)	(39.69)
γ	0.52***	0.69***	0.71***	0.38**
	(0.05)	(0.03)	(0.03)	(0.15)
Log Value	-1723.55	-1191.40	-1591.95	-679.82
LR One-sided Test	42.10***	80.88***	84.35***	22.26***

Note

*, **, and *** respectively indicate that they passed statistical tests with significant levels of 10%, 5%, and 1%; data in parentheses are standard errors.

From the results in the table, it can be seen that the LR one-sided test values all pass the significance test, indicating that there is a significant relationship between environmental variables and input factor slack variables. σ^2^ all is significantly non-zero, indicating that the model fits well. Therefore, it suggests that excluding external environmental factors and random errors in measuring TFEE is necessary. In addition, the regression coefficients of environmental variables on input factor slack variables pass the significance test, indicating that external environmental factors significantly affect input factors. Among them, a negative regression coefficient means that an increase in the value of environmental factors leads to a decrease in the redundancy of input factors. In contrast, a positive regression coefficient means that an increase in the value of environmental factors leads to an increase in the redundancy of input factors. The regression results of environmental factors are analyzed as follows:

1. Level of economic development: There is a significant positive relationship between the level of economic development in the western region for the slack variables of capital input, labor input, energy input, and Sulfur dioxide input. It indicates that an increase in the level of economic development leads to an increase in the redundancy of capital input, labor input, and energy input. The increase in the level of economic growth will promote the overall development of the region, which usually enhances the efficiency of input factor use and reduces redundancy. However, due to the limitation of technology level and management experience, the economic development in the western region is not matched with the corresponding infrastructure and industrial structure, so it increases the redundancy of input factors and negatively affects the improvement of energy efficiency.
2. Industrial structure: There is a significant negative relationship between the slack variables of the industrial structure of the western region for capital input and labor input, a significant positive relationship for energy input, and a correlation with Sulfur dioxide input that fails the statistical test. It indicates that the increase in the share of the secondary industry will reduce the redundancy of capital and labor input factors and increase the redundancy of energy input. The secondary industries in the western region are mainly resource-intensive industries, with fewer high-tech industries and other industries, and the secondary industries are primarily located at the low end of the industrial chain. Due to the limitation of the technology level, the development of secondary industries will bring energy redundancy, which could be more conducive to energy efficiency improvement.
3. Energy consumption structure: The structure of energy consumption in the western region has a significant positive effect on the slack variables of capital and energy inputs, while it has a significant negative correlation with labor inputs, and the correlation with sulfur dioxide inputs does not pass the statistical test. It indicates that the increase in the share of coal consumption increases the redundancy of capital and energy inputs while decreasing the redundancy of labor inputs. The reason may be that coal occupies a significant position in China’s energy sources, and its consumption in production has a specific substitution effect on labor, while coal use increases the additional consumption of capital and energy due to the relative backwardness of technology management in the western region.

### Stage Ⅲ: Adjusted DEA empirical results

The input factors are adjusted according to the regression results obtained in the second stage. In this paper, using Deap2.1 software, the adjusted input factors and the original output factors are again substituted into the BCC model for measurement to obtain the efficiency values of each decision unit in the third stage. Table 5 below shows the adjusted total factor energy efficiency of eleven provinces in China’s western region from 2006 to 2021.

**Table 5:** Measured TFEE of the third stage in the western region by province, 2006–2021.

Year	Inner Mongolia	Guangxi	Chongqing	Sichuan	Guizhou	Yunnan	Shaanxi	Gansu	Qinghai	Ningxia	Xinjiang
2006	1.000	0.791	1.000	1.000	0.534	0.780	0.958	0.593	0.285	0.290	0.959
2007	1.000	0.784	1.000	1.000	0.553	0.747	0.947	0.569	0.269	0.295	0.891
2008	1.000	0.772	1.000	1.000	0.583	0.726	0.996	0.537	0.277	0.318	0.839
2009	1.000	0.768	1.000	1.000	0.601	0.736	0.973	0.509	0.251	0.310	0.731
2010	1.000	0.762	1.000	1.000	0.598	0.722	0.970	0.519	0.266	0.324	0.677
2011	1.000	0.717	1.000	1.000	0.630	0.746	0.939	0.515	0.253	0.317	0.649
2012	0.956	0.684	1.000	1.000	0.684	0.786	0.936	0.510	0.250	0.312	0.642
2013	0.907	0.698	1.000	1.000	0.748	0.848	0.924	0.517	0.258	0.315	0.654
2014	0.846	0.698	1.000	1.000	0.803	0.855	0.888	0.509	0.261	0.317	0.655
2015	0.802	0.700	1.000	1.000	0.844	0.832	0.801	0.461	0.269	0.314	0.597
2016	0.742	0.641	1.000	1.000	0.821	0.790	0.800	0.431	0.276	0.307	0.550
2017	0.689	0.611	1.000	1.000	0.830	0.789	0.792	0.411	0.276	0.328	0.552
2018	0.694	0.685	1.000	1.000	0.862	0.835	0.811	0.445	0.299	0.361	0.608
2019	0.686	0.642	1.000	1.000	0.857	0.847	0.803	0.463	0.302	0.379	0.608
2020	0.650	0.590	1.000	1.000	0.851	0.829	0.759	0.471	0.297	0.401	0.586
2021	0.727	0.590	1.000	1.000	0.822	0.801	0.771	0.489	0.304	0.432	0.611
Average Value	0.856	0.696	1.000	1.000	0.726	0.792	0.879	0.497	0.275	0.333	0.676
Sequence	4	7	1	1	6	5	3	9	11	10	8

The measurement results show that after excluding external environmental factors and random interference, the average value of TFEE in western China from 2006 to 2021 is 0.721. Specifically, the comprehensive efficiency of Chongqing is 1, which is still in the efficiency frontier surface after removing external factors, while all other regions have room for improvement. Among them, the energy efficiency of Sichuan, Inner Mongolia, Shaanxi, Yunnan, and Guizhou is greater than the average value of the Western region, while Xinjiang, Guangxi, Gansu, Ningxia, and Qinghai are below the regional average, with more room for improvement.

Table 6 above shows the results of the third stage efficiency decomposition of 11 provinces in the western region from 2006–2021. There are some changes compared with the first stage, the mean value of pure technical efficiency increases to 0.934, while the mean value of scale efficiency decreases to 0.776. It shows that external environmental factors harm pure technical efficiency, which makes the first stage underestimate pure technical efficiency. The positive impact on scale efficiency makes scale efficiency overestimated in the first stage. Specifically, Chongqing is on the efficiency frontier side in both pure technical efficiency and scale efficiency, while Sichuan and Qinghai are on the efficiency frontier side in pure technical efficiency, except for the rest of the provinces, where there is room for improvement. Among them, Gansu, Qinghai, and Ningxia have more room for improvement in scale efficiency.

**Table 6:** Decomposition results of TFEE in the third stage in western provinces.

Region	Comprehensive Efficiency	Pure Technical Efficiency	Scale Efficiency	Compensation for Size
Inner Mongolia	0.856	0.951	0.896	irs
Guangxi	0.696	0.971	0.717	irs
Chongqing	1.000	1.000	1.000	-
Sichuan	1.000	1.000	1.000	drs
Guizhou	0.726	0.986	0.738	irs
Yunnan	0.792	0.951	0.835	irs
Shaanxi	0.879	0.950	0.925	irs
Gansu	0.497	0.927	0.537	irs
Qinghai	0.275	1.000	0.275	irs
Ningxia	0.275	1.000	0.275	irs
Xinjiang	0.676	0.912	0.737	irs
Average Value	0.697	0.968	0.721	

**Fig 2:**
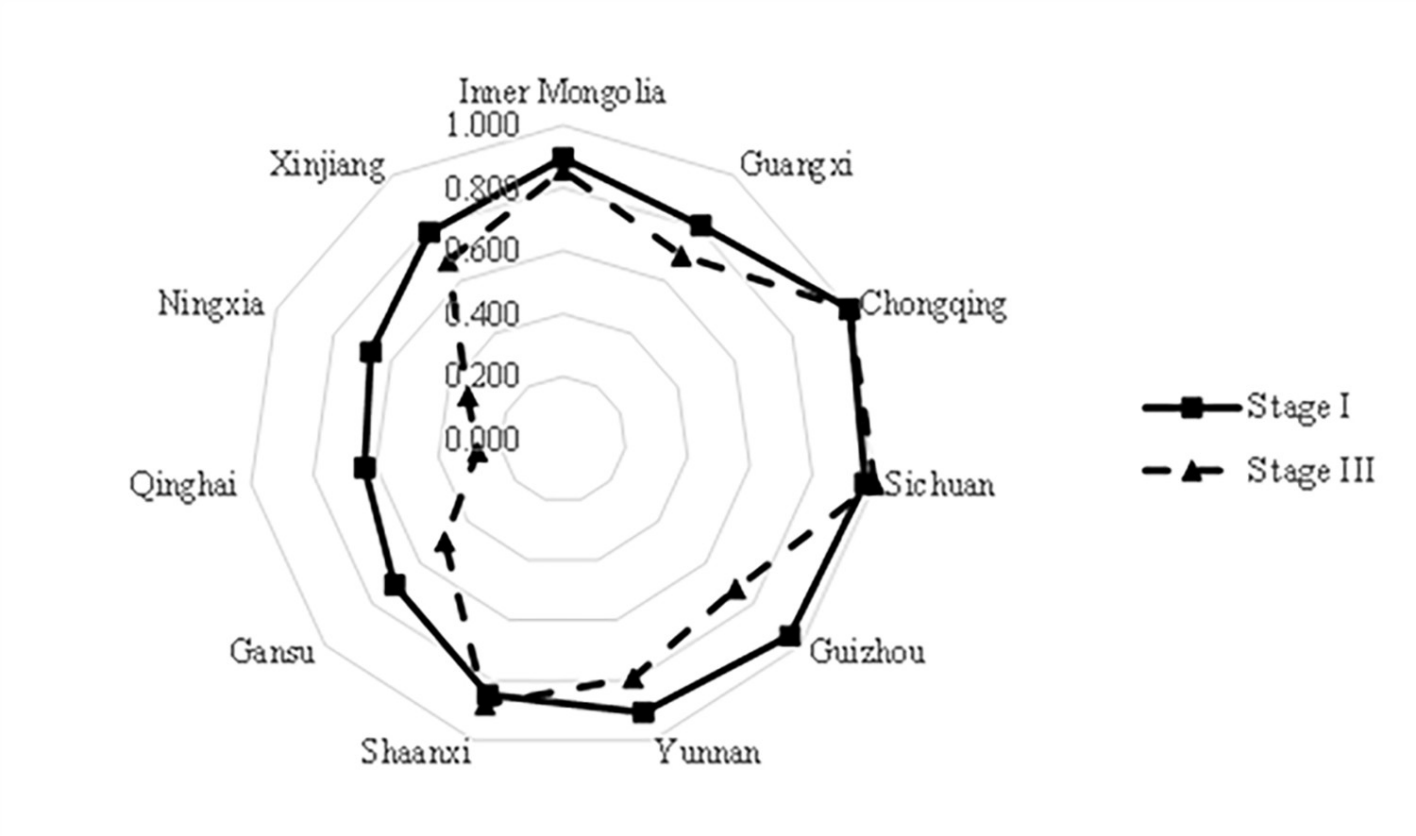
Comparison of TFEE stage Ⅰ and stage Ⅲ results by provinces in the Western region.

The total factor energy efficiency measured in the third stage is compared with the results of the first stage, as shown in Fig 2. The average integrated efficiency in the western region decreases from 0.822 to 0.721, indicating that environmental factors and random interference effects significantly impact TFEE, and the integrated efficiency in the first stage is overestimated. Specifically, the comprehensive efficiency of each province has also changed differently. Inner Mongolia, Guangxi, Chongqing, Guizhou, Yunnan, Gansu, Qinghai, Ningxia, and Xinjiang all have different degrees of decline after adjustment, indicating that the efficiency measured in the first stage is affected by the external favorable factors in which they are located. Among them, Guizhou, Gansu, Ningxia, and Qinghai have a relatively significant decline, indicating that their favorable external environment is caused by the impact of the larger. The efficiency of Sichuan and Shaanxi increased after the adjustment. Still, they were weak, indicating that the efficiency of these provinces in the first stage was negatively affected by the soft external environmental factors.

## Exploration of the factors influencing TFEE in the western region based on the Tobit model

### Model construction and variable description

The previous paper measures the total factor energy efficiency excluding external factors and random interference, which is more accurate than the first stage. In this paper, the factors affecting TFEE can be divided into external and internal factors. External factors, such as the environmental factors excluded from the second stage SFA regression above, cannot be improved in a short period, while internal factors can be improved through policies and other regulations. To improve TFEE, the influence of internal factors should be further explored.

Based on the above, this paper selects the indicators of TFEE in the third stage as the explanatory variables and the level of science and technology innovation, environmental regulation intensity, and the level of external openness as the explanatory variables concerning previous studies and data availability. The variables are described as follows:

1. The level of science and technology innovation: Science and technology is the first productive force. Enterprises need to continuously improve their knowledge-sharing ability, absorption ability, and knowledge innovation ability so as to enhance green knowledge collaboration ability [25]. Science and technology innovation can improve technical efficiency, which in turn can promote the improvement of energy efficiency. This paper adopts the proportion of industrial enterprises’ research and experimental development (R&D) expenditure above the scale of the year’s GDP to measure the level of science and technology innovation.
2. Environmental Regulation Intensity: Improving energy efficiency and environmental protection relies to some extent on government support and guidance. The intensity of environmental regulation reflects the extent to which the government is guiding environmental protection. In this paper, we use the proportion of industrial pollution control costs to the total industrial output to measure the intensity of environmental regulation.
3. The level of external openness: External openness can attract foreign investment and bring technology, which affects energy efficiency to a certain extent. Improving the level of external openness can promote the advancement of energy efficiency. In this paper, we choose the proportion of total import and export trade to GDP of the year to measure the level of external openness.

However, since the TFEE obtained from the third stage DEA is restricted to the (0, 1] interval, the estimation using ordinary least squares is prone to bias, so the Tobit regression model is chosen for modeling. Tobit regression model is a widely used model in econometrics, initially proposed by James Tobin in 1958, and is mainly used to solve problems in the presence of truncated. Tobit regression model is a widely used model in econometrics. Accordingly, this paper constructs a regression model for empirical testing.

### Empirical results

This paper uses Stata 17.0 software for regression. Each variable passed a robustness test to ensure the robustness of the data before regression, and the Tobit regression results were obtained as follows:

Table 7 above demonstrates the regression results, where the variables passed the significance test, and based on the regression results, it can be concluded that

1. The level of science and technology innovation: the level of science and technology innovation has a significant positive correlation with the TFEE, with a correlation coefficient of 0.727. Technological innovation can improve energy utilization efficiency, but the level of original technological innovation in the Western region is low. However, in recent years, with the "western development", "one belt and one road" and other strategies and the transfer of industries from the eastern region, the level of science and technology innovation has been significantly improved. In recent years, with the promotion of "Western Development", "One Belt, One Road" and other strategies and the transfer of industries in the eastern region, its level of scientific and technological innovation has been significantly improved. The increase of industrial enterprises’ investment in research and experimental development can improve their technology level, which in turn can promote the improvement of energy utilization and the reduction of energy consumption of industrial enterprises, and has a strong positive impact on the progress of total factor energy efficiency.
2. Environmental regulation intensity: Environmental regulation intensity negatively correlates with the improvement of TFEE, with a correlation coefficient of -0.810. In recent years, the western region has paid more attention to environmental pollution. Still, the increase in industrial pollution control costs has increased production costs to a certain extent, affecting TFEE and, therefore, hindering the improvement to some extent.
3. The level of external openness: the level of external openness has a significant positive correlation with the TFEE, with a correlation coefficient of 0.872. Along with the import and export trade increase, the western inland region can learn advanced technology and management experience, affecting energy efficiency to a certain extent. However, since the impact of technology spillover on energy efficiency is slower and weaker than that of direct technological innovation, the correlation coefficient of the level of opening up is lower than that of technological innovation.

**Table 7:** Regression results of factors influencing TFEE in the Western region.

Variables	Coefficient	Standard Error	T-value
Constants	0.420***	0.071	5.881
The Level of Science and Technology Innovation	0.727***	0.198	3.675
Environmental Regulation Intensity	-0.810***	0.173	-4.692
The Level of External Openness	0.872***	0.182	4.784

Note

*** means passing the statistical test of 1% significance level respectively.

### Robustness test

From the results of the previous three-stage DEA decomposition, we can see that the changes in scale efficiency mainly cause the changes in total factor energy efficiency in the western region. Therefore, in this paper, the scale efficiency obtained from the third stage decomposition is used as the explanatory variable instead of the integrated efficiency, and the explanatory variables are kept consistent with the previous paper for Tobit regression.

The variables in the above table 8 pass the significance test. According to the regression results, it can be concluded that the level of science and technology innovation and the level of external openness have a significant positive effect on scale efficiency, and the intensity of environmental regulation has a significant negative impact on scale efficiency. The coefficient signs of the three explanatory variables are consistent with those of the explanatory variables in the basic regression model in the previous section, which proves that the model has certain robustness.

**Table 8:** Regression results of factors influencing total factor energy scale efficiency in the Western region.

Variables	Coefficient	Standard Error	T-value
Constants	0.464***	0.072	6.450
The Level of Science and Technology Innovation	0.730***	0.200	3.655
Environmental Regulation Intensity	-0.860***	0.173	-4.706
The Level of External Openness	0.866***	0.184	4.968

Note

*** means passing the statistical test of 1% significance level respectively.

## Discussion

The empirical results are as follows:

1. After excluding external environmental factors, the mean value of TFEE decreases, pure technical efficiency increases, and scale efficiency decreases. It indicates that environmental factors make the overall efficiency and scale efficiency overestimated and underestimate the pure technical efficiency. In addition, the provinces also show different levels of change. This is consistent with the findings of most studies, such as reference 17. It suggests that environmental factors and stochastic disturbance terms are essential in TFEE.
2. he external environmental factors in the western region significantly affect input factors. The level of economic development has a significant positive relationship with the slack variables of capital input, labor input, and energy input. Industrial structure has a meaningful negative relationship between the slack variables of capital and labor input and a significant positive connection with the slack variables of energy input. Energy consumption structure has a significant positive effect on the slack variables of capital input and energy input, while it has a significant negative correlation on labor input.
3. The regression of internal influencing factors reveals that the level of science and technology innovation and the level of external openness have a significant positive correlation to the TFEE. This is consistent with the findings of most studies, such as reference 24. However, the intensity of environmental regulation in this study shows a negative correlation with TFEE, which is inconsistent with the findings of some studies. According to Wu, environmental regulation has a U-shaped relationship with TFEE, and excessive environmental regulation intensity will hurt TFEE [26]. Therefore, this paper argues that the current intensity of environmental regulation in the western region has a mismatch with its development, which leads to the negative impact of its environmental regulation intensity on TFEE. Meanwhile, the regression results using scale efficiency instead of comprehensive efficiency as the explanatory variables remain consistent, proving the robustness of the above regression.

## Conclusions and future prospects

### Conclusions

Based on the above conclusions, this paper proposes the following countermeasures for the Western region to promote the "double carbon" goal, industrial upgrading, and sustainable economic development:

1. Considering external factors, it is important to consider how environmental factors affect TFEE. It is crucial to utilize favorable external factors while avoiding the negative impact of unfavorable ones. Different regions should take into account their unique environmental conditions and adjust their industrial development plans accordingly. This will help improve energy efficiency and promote sustainable economic development.
2. Considering internal factors, businesses should prioritize scientific and technological innovation, incorporate advanced technologies and skilled professionals, invest in developing new products and technologies, and elevate their position in the industrial chain. It is also essential for companies to collaborate with the "One Belt, One Road" initiative to increase their global presence, acquire cutting-edge technology and management expertise, and expand foreign trade. Firms should implement tailored environmental protection policies and comply with appropriate environmental regulations to accommodate regional industrial characteristics. Enterprises need to continuously improve their knowledge-sharing ability, absorption ability, and knowledge innovation ability so as to enhance green knowledge collaboration ability。

### Limitations

Overall, this paper still has some things that could be improved. For the research object, this paper selects the upgrading data in western China and does not select the microdata of prefecture-level cities. We will collect prefecture-level city data for further exploration in the subsequent study. For the research method, this paper uses a three-stage DEA model to measure TFEE. Still, in terms of considering undesired outputs, it simply uses the method of considering undesired outputs as inputs without changing the structure of the production possibility set. In the subsequent research, we will innovate and further explore the three-stage DEA model for undesired factors.
